# A dataset of healthcare road accessibility in Australia: service-based grouping and residential reachability

**DOI:** 10.1016/j.dib.2025.112068

**Published:** 2025-09-12

**Authors:** Kiki Adhinugraha, Thanh Phan, Richard Beare, Albert Phan, Shiyang Lyu, David Taniar

**Affiliations:** a Department of Computer Science and Information Technology, La Trobe University, Australia; b Department of Neurology, Monash Health, Australia; c School of Clinical Sciences at Monash Health, Faculty of Medicine, Monash University, Australia; d National Centre for Healthy Ageing, Peninsula Clinical School, Faculty of Medicine, Monash University, Australia; e Developmental Imaging, Murdoch Children's Research Institute, Melbourne, Australia; f Department of Medicine, University of Melbourne; g Faculty of Information Technology, Monash University, Australia; h Victorian Heart Institute, Monash University, Australia

**Keywords:** Healthcare accessibility, Driving distance, Hospital group, Clinical services, Public health planning

## Abstract

This dataset provides national coverage of healthcare road accessibility in Australia, focusing on five types of facilities: four hospital groups based on clinical service availability and one group of ambulance stations. Hospitals are classified into four groups based on the number of clinical services they offer: Blue (>16 services), Green (11–15), Orange (5–10), and Red (<5). Ambulance stations are treated as a distinct group to support emergency access analysis. For each group, the dataset presents travel distance and estimated driving time from major road points, enabling a consistent and structured view of spatial access to healthcare services across the country.

Road accessibility is calculated using shortest-path routing across OpenStreetMap road networks. Estimated travel times are adjusted based on road speed limits and regional driving conditions (urban, regional, or remote). Two accessibility layers are provided: (1) a comprehensive set of road nodes showing distance (in kilometres) and travel time (in hours) to the nearest hospital in each group, and (2) a set of sampled residential address points, representing population perspectives, with linked nearest-healthcare metrics for each group, and aggregated summaries by SA2, LGA, and remoteness levels.

Travel time accuracy was validated against Google Maps API, with average discrepancies of 3 min in urban areas, 10 min in regional areas, and 90 min in remote areas. Summary statistics are also available at the Local Government Area (LGA), Statistical Area Level 2 (SA2), and remoteness levels, allowing high-level regional comparisons.

The dataset includes vector-based geospatial files representing hospitals, ambulance stations, road nodes, and residential samples. All layers use the GDA2020 datum (EPSG:7844) and are derived from the Australian Institute of Health and Welfare (hospital services), Digital Atlas Australia (ambulance locations), OpenStreetMap (road networks), Australian Bureau of Statistics (boundaries), and Geoscape G-NAF (residential addresses).

The dataset supports public health planning by illustrating which facilities are reachable by road, the estimated distance and travel time to reach them, and how hospital service capacity influences spatial accessibility.

Specifications Table

**Table:** 

Subject	Health Sciences, Medical Sciences & Pharmacology
Specific subject area	Australian hospital accessibility dataset using road-network-based catchments, travel time modeling, and real residential addresses for GIS and health planning
Type of data	Table (CSV), Filtered (CSV), Processed (CSV), Geospatial Vector layer (GeoJSON)
Data collection	Hospital data, including locations and clinical services, were obtained from AIHW and manually verified using Google Maps. Hospitals were grouped into four categories based on service capacity. Ambulance station locations were sourced from Digital Atlas Australia and verified. Administrative boundaries, including LGA and Remoteness classifications, were obtained from ABS, with Remoteness simplified into three groups (Cities, Regional, Remote). Road data were sourced from OSM, using Highway, Trunk, and Primary roads. 20k residential addresses (G-NAF) were sampled from Statistical Area Level 2, selecting one per residential mesh block. Network Voronoi Diagram (NVD) estimated travel distances and times for hospital and ambulance catchments. LGA and Remoteness-level aggregations summarize accessibility metrics by locality and remoteness. The dataset was validated using Google Maps API, comparing computed travel times with real-world estimates.
Data source location	Hospital Services and Locations: https://www.aihw.gov.au/reports-data/myhospitals/content/data-downloads/hospital-downloads Ambulance Stations: https://digital.atlas.gov.au/datasets/323bff87ddc944d1938c2fcc006d5707_0/explore?location=−26.423203%2C137.139041%2C4.69 Australia Administrative Boundaries: https://www.abs.gov.au/statistics/standards/australian-statistical-geography-standard-asgs-edition-3/jul2021-jun2026/access-and-downloads/digital-boundary-files Australia Remoteness Boundaries: https://www.abs.gov.au/statistics/standards/australian-statistical-geography-standard-asgs-edition-3/jul2021-jun2026/remoteness-structure/remoteness-areas
	Australia Road Data: https://download.geofabrik.de/australia-oceania/australia.html Australia Residential Addresses: https://data.gov.au/dataset/ds-dga-19432f89-dc3a-4ef3-b943–5326ef1dbecc/details
Data accessibility	**Please note:** All raw data referred to in this article must be made publicly available in a data repository prior to publication. Please indicate here where your data are hosted (the URL must be working at the time of submission and editors and reviewers must have anonymous access to the repository): Repository name: A Dataset of Healthcare Road Accessibility in Australia: Service-Based Grouping and Residential Reachability Data identification number: Not Applicable Direct URL to data: https://doi.org/10.6084/m9.figshare.26790337.v1
Related research article	None.

## 1 Value of the Data

• **National dataset of healthcare road accessibility in Australia**
  This dataset provides high-resolution road-based accessibility data for public hospitals and ambulance stations across Australia. It includes travel distance (in kilometres) and estimated time (in hours) from any road point to the nearest facility within each service-based group.
• **Hospitals grouped by clinical service availability**
  Public hospitals are grouped into four categories based on the number of clinical services offered. This enables targeted analysis of healthcare access across different levels of service capacity, from basic to highly specialised care.
• **Realistic road-based accessibility measures**
  Accessibility metrics are calculated using shortest-path analysis on the road network, incorporating speed limits and regional speed penalties. Each record includes estimated driving distance (km) and time (hr) to the nearest healthcare facility by group, supporting realistic, high-resolution spatial analysis.
• **Supports a wide range of applied research and planning applications**
  Application layers are included to demonstrate practical use of the accessibility data. These include residential address mapping, which links 20,000 real addresses to their nearest healthcare facilities, and regional summaries at the SA2, LGA, and remoteness levels. These applications illustrate how the dataset can support studies in healthcare access, service equity, and transport planning at both household and policy levels.

## 2 Background

Life expectancy is a widely recognised measure of population health, shaped by factors such as age, socioeconomic status, and access to healthcare. In Australia, research by the Australian Institute of Health and Welfare (AIHW) highlights a clear disparity in life expectancy between urban and remote regions. Between 2018 and 2022, the age-standardised death rate in Very Remote areas was 770 deaths per 100,000 population—1.6 times higher than in Major Cities (492 deaths). For specific conditions, the gaps are wider: diabetes caused 54 deaths per 100,000 in Very Remote areas versus 15 in Major Cities (3.2 × higher), and coronary heart disease accounted for 97 versus 49 deaths (2.0 × higher) [1].

One of the critical contributors to this gap is access to medical facilities, yet many existing studies rely on straight-line distances rather than actual travel conditions, leading to oversimplified assessments of healthcare accessibility. For example, Barbieri and Jorm [2] estimated hospital accessibility using Mesh Block centroids, aggregating distances at the Statistical Area Level 2 (SA2). However, this method assumes that all hospitals provide the same level of service and does not account for uninhabited areas, potentially misrepresenting true accessibility. In reality, only 103 out of 695 public hospitals in Australia have coronary care units [3], meaning that patients requiring specialised cardiac care may need to travel significantly farther than general proximity-based models suggest.

To address these limitations, this dataset introduces a group-based approach to model road accessibility to healthcare facilities. Hospitals are categorised into four groups based on the number of clinical services they provide, allowing a clearer distinction between general and specialised care. Ambulance stations are treated as an additional group, enabling direct comparison of access to emergency response points. Road accessibility is calculated using realistic driving conditions, factoring in speed limits and regional speed penalties across urban, regional, and remote areas [9].

Additionally, 20,000 real residential addresses were sampled across the country and linked to their nearest hospitals in each group. This approach provides high-resolution insights into how accessible different levels of care are, especially in underserved areas.

By focusing on road-based accessibility rather than theoretical proximity, this dataset helps researchers, planners, and policymakers better understand healthcare access across Australia. It supports data-driven decisions in emergency preparedness, service delivery, and regional health planning.

## 3 Data Description

This dataset presents geospatial and non-spatial layers that describe road-based access to healthcare services across Australia. It estimates travel distances and times to the nearest facility within each service-based hospital group using actual road segments. These accessibility layers are grouped by the number of clinical services available at each facility, allowing comparisons across hospital capacity tiers or ambulance station.

The dataset is organised into two categories: (1) healthcare and road accessibility layer, and (2) application layer. All spatial layers use GDA2020 (EPSG:7844) for national consistency. he full list of files, including format and description, is provided in metadata.txt.

### 3.1 Healthcare and accessibility layers

This group forms the core contribution of the dataset. It includes the locations of healthcare facilities (hospitals and ambulance stations) and road-based accessibility metrics calculated for each facility group. Accessibility is modelled along actual road paths using a shortest-path algorithm with speed penalties based on road type and remoteness.

To ensure consistency and practicality at a national scale, the road network used in this computation is limited to major road types only—specifically highways, trunks, and primary roads, as defined in OpenStreetMap. Lower-order roads such as residential streets, service lanes, and private access ways were excluded, as they introduce excessive noise and have limited impact on inter-regional healthcare access.

• Hospital and ambulance locations are provided in hospital.geojson and ambulance.geojson, including coordinates, names, number of clinical services (for hospitals), and classification group (for hospital - see Table 1).
• Edges files (e.g., hospital_blue_edges.geojson) represent road segments leading to the nearest facility within each group.
• Nodes files (e.g., hospital_blue_nodes.geojson) contain estimated travel distance (km) and time (hours) from each road node to the nearest facility in that group. These values are used for further mapping and aggregation.

**Fig. 1:**
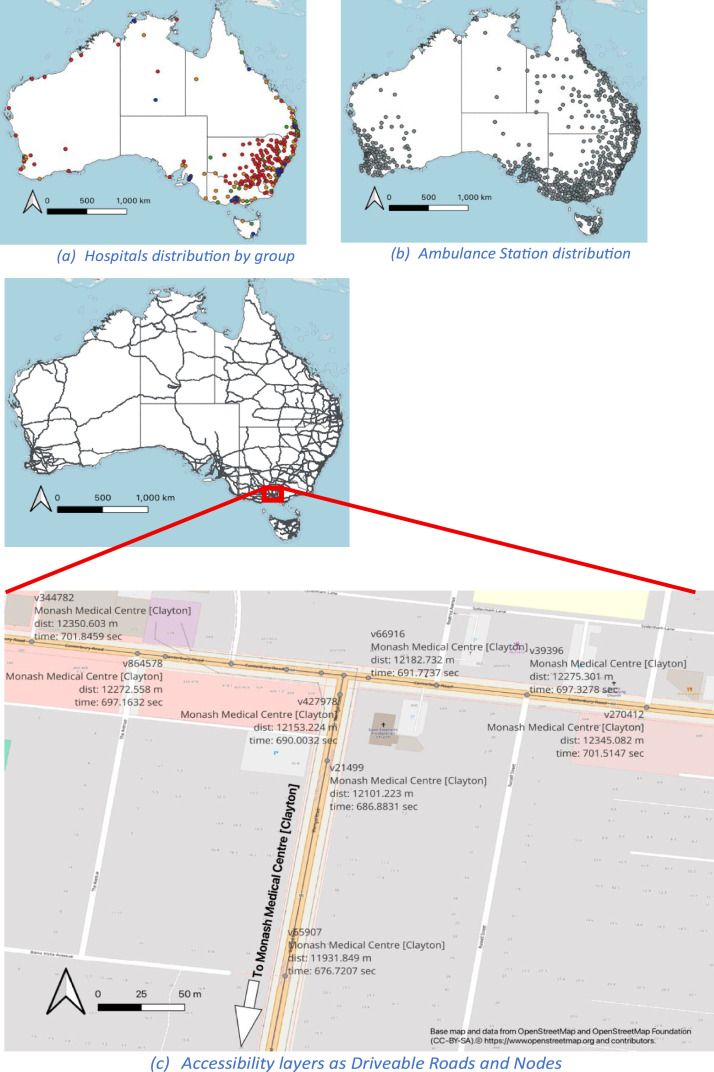
Healthcare and Road Accessibility Data.

The healthcare road accessibility layers is shown in Fig. 1. In addition to static maps, users can explore these layers interactively through a publicly accessible Shiny application. The application can be accessed using this link https://kiki-maulana.github.io/road_accessibility/ .

### 3.2 Application layers

These layers demonstrate how the accessibility data can be applied to evaluate real-world access from residential locations and support high-level regional comparisons. There are two types of application layers, which are:

• Residential Access Mapping. This dataset demonstrates how to perform household-level access analysis, offering a more realistic measure than areal centroids or mesh block approximations. The dataset includes sample_addresses.geojson that contains 20,000 geocoded residential points sampled from Geoscape’s G-NAF database, and address_nearest_facility.csv, which links each sampled address to its nearest hospital in each group and ambulance station, with estimated travel distance and time.
• Aggregated Data by Region. This dataset provides summary statistics at three different level. To support national comparability, it adopts standard geographic classifications from the Australian Bureau of Statistics (ABS), including Statistical Area Level 2 (SA2), Local Government Area (LGA), and a simplified Remoteness classification based on the 2021 ASGS. Only the remoteness boundary layer is included directly; other official boundary files can be accessed via the ABS [4].
• Each summary provides the average distance, time, and the most common nearest facility (by group) within the region. In areas lacking data coverage (due to low population or no qualifying roads), values are interpolated from adjacent regions. A separate interactive dashboard is available at https://kiki-maulana.github.io/road_accessibility/, which allows users to explore these aggregated patterns through maps

## 4 Experimental Design, Materials and Methods

**Fig. 2:**
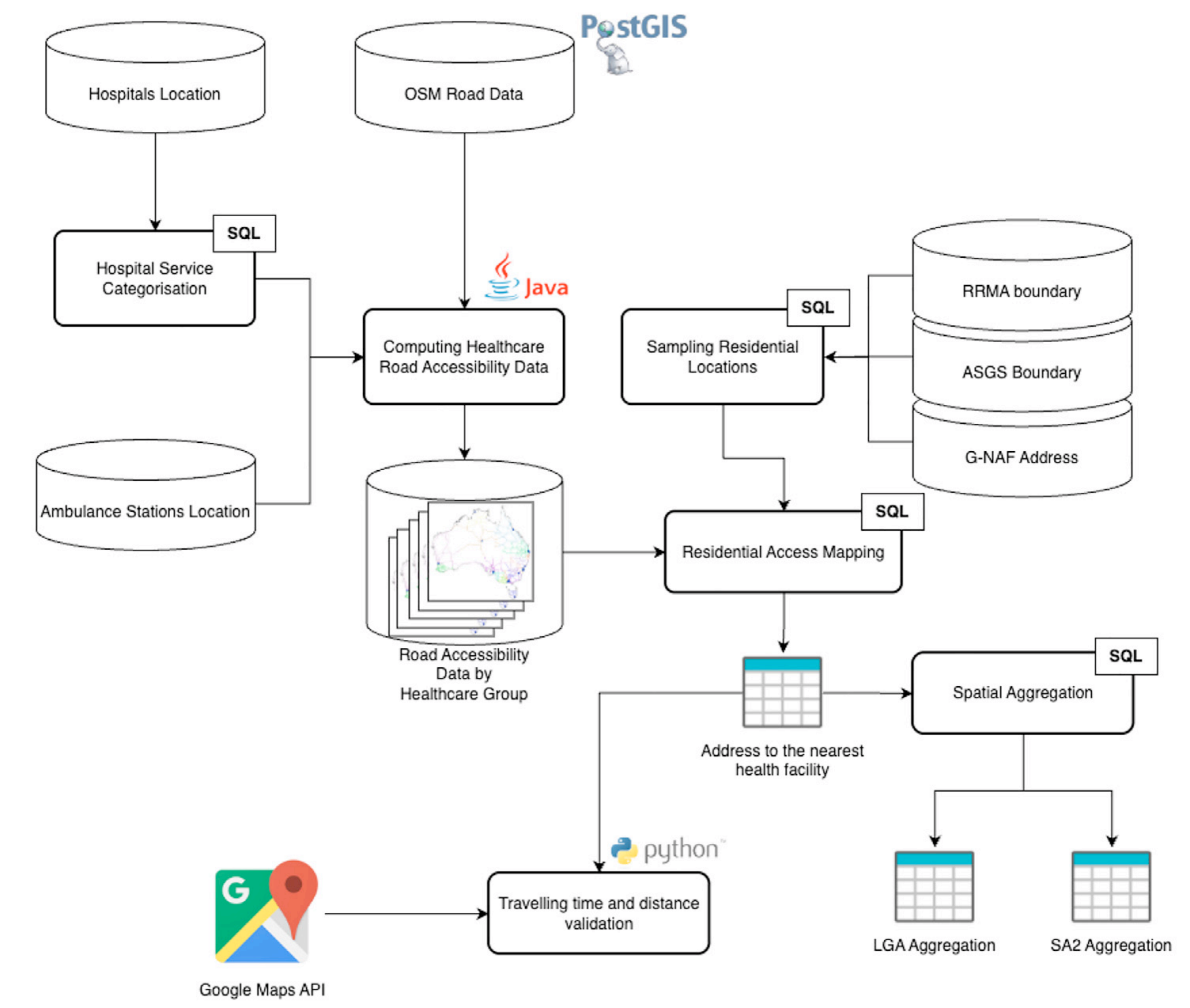
Healthcare Road Accessibility processing framework.

This section describes the end-to-end process used to produce the healthcare road accessibility dataset for Australia. The workflow, illustrated in Fig. 2, involves six key stages: (1) acquiring and preparing national datasets, (2) categorising hospitals by their clinical service capacity, (3) computing road-based accessibility to hospitals and ambulance stations, (4) mapping residential access to nearby facilities, (5) validating estimated travel times, and (6) aggregating results by standard administrative boundaries. The catchment identification process was designed to generate service areas based on realistic travel conditions. Instead of using straight-line distances, accessibility is modelled along actual road segments, accounting for speed limits, regional travel penalties, and hospital service availability—offering a more accurate and practical view of access to healthcare.

All spatial data are restored in a PostgreSQL 12.4 database with PostGIS 3.0 extension, where SQL processing and aggregation are conducted. Road accessibility computations are performed using NetBeans 12 with Java SE Runtime Environment 16.0. The travel time validation from a residential address to the nearest healthcare facility is performed using Python scripts with the Google Maps API. Spatial visualisations are created in QGIS 3.40. All processing was conducted on a macOS 15.6 machine (M2, 16 GB RAM). To support data exploration, a set of interactive web applications was developed using R Shiny. These applications allow users to visualise road accessibility and perform regional comparisons. The apps are currently hosted on a cloud platform for public access.

### 4.1 Data acquisition and preparation

The development of this dataset relies on the integration of multiple authoritative sources to ensure national coverage and consistency. There are four types of data sources, which are the Australian healthcare facilities, roads, boundaries, and residential addresses.

This dataset includes two types of healthcare locations: hospitals and ambulance stations. Hospital data, including their service types, were sourced from the Australian Institute of Health and Welfare (AIHW) [5], filtered to include only hospitals with emergency departments that serve at least 250 patients. Ambulance station locations were obtained from Digital Atlas Australia [13], with manual validation using OpenStreetMap (OSM) and Google Maps to ensure accuracy, particularly for stations located in remote or unpopulated areas.

Road network data were sourced from OpenStreetMap, downloaded on 25 February 2023 [6], focusing exclusively on major transport routes—highways, trunk roads, and primary roads. Lower-level streets (residential, service, and unclassified roads) were excluded to prioritise routes that reflect realistic interregional access. The resulting network consists of 139,536 road segments and 944,771 road nodes. This ensures that estimated travel paths reflect feasible driving routes for emergency and public access to healthcare facilities.

Three boundary layers were used from the Australian Bureau of Statistics (ABS): Statistical Areas Level 2 (SA2), Local Government Areas (LGA), and Remoteness Areas. SA2 boundaries are part of the ABS Statistical Structure and represent socially and economically linked communities. They offer a balance between detail and manageability, making them suitable for healthcare analysis. LGA belong to the ABS Non-Statistical Structure and reflect jurisdictional administrative boundaries defined by state and territory governments. The ACT is treated as a single unit due to its lack of LGAs. Remoteness Areas classify regions based on relative access to services, using the Accessibility and Remoteness Index of Australia (ARIA+) [8]. The original classification includes five categories—Major Cities, Inner Regional, Outer Regional, Remote, and Very Remote—but in this dataset, a simplified classification is applied, grouping all locations into three categories: City, Regional, and Remote. This simplified structure supports more intuitive analysis while preserving essential distinctions in access characteristics.

All boundary layers are derived from the Australian Statistical Geography Standard (ASGS) Edition 3 [4] and use the GDA2020 coordinate reference system (EPSG:7844). Only the simplified Remoteness boundary is included in the dataset; the full boundary files for SA2 and LGA are publicly available from the ABS and can be accessed as needed for extended analyses.

To improve the realism of access estimates, this study uses a sample of 20,000 geocoded residential addresses from the Geoscape G-NAF (Geocoded National Address File) [7]. Unlike studies that rely on population centroids or Mesh Block averages, this sampling approach calculates access from actual residences, supporting higher-resolution evaluations of service reach and spatial disparities.

### 4.2 Hospital service categorisation

To support meaningful comparisons of healthcare accessibility, hospitals in this dataset were grouped based on the number of clinical services they provide, rather than relying on AIHW’s official peer group classification system. While the AIHW peer groups offer detailed role-based categories across 36 types [12], these are often too granular and inconsistent for spatial accessibility analysis — for example, large hospitals with broad service coverage may still fall into different peer groups due to administrative criteria.

Instead, this study presents a simplified service-based grouping by counting the total number of clinical services at each hospital. This approach allows for a more objective comparison of healthcare capacity and better reflects the range of services available to local populations. To ensure quality and relevance, the dataset includes only public hospitals that have an emergency department and serve at least 250 emergency presentations annually. Hospitals with missing service information were excluded from the classification process.

**Fig. 3:**
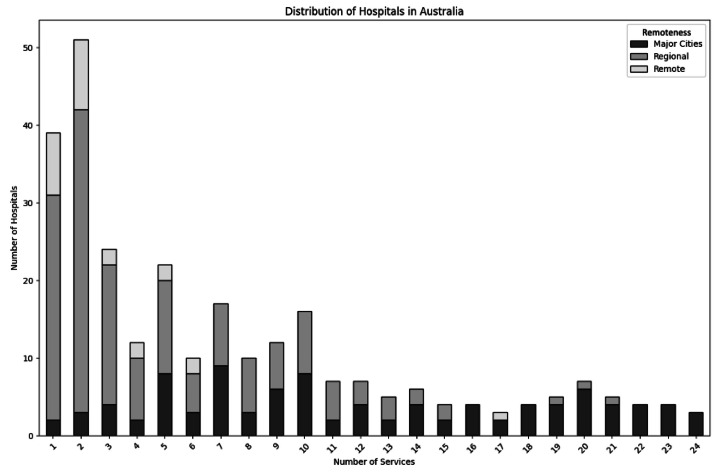
Hospital distribution by number of services.

The AIHW dataset includes up to 24 clinical service types per hospital. Analysis of this distribution shows a clear right-skew: most hospitals—especially in regional and remote areas—offer fewer than 10 services, while only a small number (mostly metropolitan) offer >16. Based on this distribution, hospitals were grouped into four categories, designed to balance detail with interpretability, as shown in Table 1. The distribution of hospitals by service count is shown in Fig. 3.

**Table 1:** Hospital categorisation based on number of clinical services.

Group	Number of Clinical Services	Description
Blue	16+	Large hospitals with extensive services, mostly found in metropolitan areas
Green	11–15	Facilities with a wide range of services, present in both regional and urban areas
Orange	6–10	Facilities with moderate services, typically in larger regional towns
Red	1–5	Hospitals with very limited services, commonly in remote or small regional areas

Colour naming was selected not only for compactness, but also for visual communication: Blue and Green signal more complete access, while Red draws attention to potential service limitations. This grouping enables tiered analysis of healthcare access by facility capability.

This grouping approach improves the interpretability of healthcare accessibility by aligning the hospital service range with common regional classifications, without making assumptions about the specific types of services each facility offers. It provides a practical framework for analysing road accessibility to healthcare across different service capacities and geographic contexts.

### 4.3 Computing healthcare road accessibility data

To model healthcare road accessibility, we applied a nearest-facility allocation approach based on travel distance and time along actual roads. This was implemented using a custom Java application that computes the shortest path from each road node to the nearest health facility, using a time-based Network Voronoi Diagram (NVD) algorithm [9] to calculate the estimated travelling distance and time. The speed penalty or reduction concept is applied based on the speed limit and remoteness to simulate average congestion. Unlike traditional proximity models that rely on straight-line distances, this method incorporates road topology and travel conditions, resulting in more realistic estimates of service reachability.

The analysis was performed on OSM road data [6]. The final road dataset includes 139,536 road segments and 944,771 nodes for each healthcare group. The road segments (edges) represent the accessible road paths leading to the nearest facility in the group, while the road nodes (points) store incremental and accumulated travel metrics, including distance (in metres) and time (in seconds), as well as a via attribute indicating the preceding node in the shortest path sequence.

Each road segment and node is assigned to a specific hospital or ambulance station based on the shortest travel path, allowing for precise delineation of accessibility zones. The use of accumulated travel time in seconds ensures accuracy in temporal estimation, especially in densely connected areas.

Interactive web applications to visualise the healthcare and the road accessibility map is provided using Shinyapps that can accessed here https://kiki-maulana.github.io/road_accessibility/.

### 4.4 Residential access mapping

This method aims to evaluate healthcare accessibility from a residential perspective by estimating travel distance and time from real addresses to the nearest health facility in each group. Although local residential roads are excluded from the road accessibility model, each address can still be mapped to the nearest major road node—a point along the higher-order road network used in the accessibility computation. These nodes store precomputed driving distance (in meters) and time (in seconds) to the nearest Blue, Green, Orange, and Red hospitals, as well as to the closest ambulance station.

Because node identifiers are consistent across all groups, identifying the nearest node to an address allows for quick retrieval of travel estimates to all facility groups. This method enables high-resolution, address-level assessment of healthcare access using a lightweight and consistent approach compatible with national-scale analysis.

### 4.5 Travelling distance and time validation

To assess the realism of the estimated travel distances and times in this dataset, a validation exercise was conducted on 13 March 2023 using on-demand queries via the Google Maps Distance Matrix API [10,11]. A representative subset of 3609 addresses—approximately 18 % of the total 20,000 samples—was selected to ensure geographic diversity across urban, regional, and remote areas.

For each selected address, driving distance and travel time to the nearest healthcare facility in each group were retrieved from Google Maps and compared with the corresponding precomputed values in the dataset. Our model estimates are based on static road segment lengths and posted speed limits, with adjustments for road class and regional penalties. In contrast, Google Maps generates dynamic estimates that incorporate real-time traffic and historical patterns, leading to potential variations.

In compliance with the Google Maps Platform Terms of Service, these comparisons were used solely for internal verification and are not included in the published dataset or distributed outputs. The comparison was conducted using a Python script and was limited due to API usage quotas and associated costs.

The summary of validation results is presented in Table 2. These tables show the average travel time differences (in minutes) between our precomputed model and Google Maps estimates, grouped by remoteness category and distance range. While travel distances remained broadly consistent between this dataset and Google Maps, travel time estimates diverged more noticeably with increasing remoteness. On average, differences were approximately 3 min in urban areas, 10 min in regional areas, and up to 90 min in remote areas. These variations highlight the challenges of modelling travel in less connected regions and underscore the importance of incorporating local road characteristics—such as road type, surface conditions, and route availability—when estimating healthcare accessibility.

**Table 2:** Time differences between the precomputed model and Google Maps by remoteness group.

Remoteness	Time Differences (minutes)	Population
City	3.12	2124
Regionals	10.66	1370
Remotes	90.09	115
**Overall**	**–**	**3609**

### 4.6 Spatial aggregation

To support regional and national-scale analysis, healthcare road accessibility data was aggregated across three spatial units: Local Government Areas (LGA), Statistical Areas Level 2 (SA2), and Remoteness Areas using SQL script. For each unit, the nearest hospital or ambulance station in each group was identified based on the residential sample points and corresponding nearest road nodes. The average travel distance (in kilometres) and time (in hours) were then computed by combining node-level metrics and sampled address mappings.

In some cases, a region might be equally close to multiple hospitals within the same group. When this occurs, all relevant hospitals are listed as the nearest options. This highlights areas where residents have more than one choice, especially for access to higher-service hospitals like Blue or Green group hospitals.

In other situations, a region may not contain any sampled addresses or relevant roads used in the accessibility analysis. This can happen in places with very few residents or where only local residential streets exist, which were not included in the road accessibility calculations. For these cases, the average travel distance and time from neighbouring areas were used, and the nearest facilities were drawn from surrounding regions.

To enhance usability, an interactive web application has been developed to explore the aggregated data. Users can view average accessibility metrics and nearest healthcare facilities across SA2, LGA, and Remoteness classifications. The application is available at https://kiki-maulana.github.io/road_accessibility/.

## Limitations

This study has several limitations that should be considered. Only major roads (highways, trunk roads, and primary roads) were included in the catchment model due to processing constraints in handling Network Voronoi Diagram (NVD) structures at scale. As a result, local and residential roads were excluded, which may impact last-mile accessibility calculations.

The validation process was limited by Google Maps’ policy restricting large-scale data scraping. Due to this constraint, only 3609 addresses were validated, reducing the ability to assess model accuracy across all regions. Additionally, the travel time estimates are static and do not account for dynamic road conditions such as congestion, construction, or road closures, which may cause discrepancies between predicted and real-world travel times.

Some high-density road areas, such as urban cores and transport hubs, may require further refinement in precomputed models to improve accuracy. Furthermore, hospital service availability is subject to change, meaning that periodic dataset updates would be necessary to reflect new hospitals, service expansions, or closures over time. Addressing these limitations in future work would enhance the dataset’s accuracy and applicability for long-term healthcare accessibility analysis.

## Ethics Statement

The authors confirm that they have read the ethical requirements for publication in Data in Brief and confirm that the current work does not involve human subjects, animal experiments, or any data collected from social media platforms.

The data source and presented data are already part of the public domain under CC-BY 4.0 License.

## Credit Author Statement

**KA, TP, RB, DT:**Conceptualisation, **KA, SL, DT:** Methodology, **KA, AP:** Software, **TP, RB, DT:** Validation, **KA, SL, DT:** Formal Analysis, **KA, AP:** Investigation, **KA, AP:** Data Curation, **KA, SL:** Writing - Original Draft, **TP, RB, DT:** Writing – Review & Editing, **KA, AP:** Visualisation.

## Data Availability

FigshareA Dataset of Healthcare Road Accessibility in Australia: Service-Based Grouping and Residential Reachability (Original data).
